# Deterioration of autonomic neuronal receptor signaling and mechanisms intrinsic to heart pacemaker cells contribute to age‐associated alterations in heart rate variability *in vivo*

**DOI:** 10.1111/acel.12483

**Published:** 2016-05-10

**Authors:** Yael Yaniv, Ismayil Ahmet, Kenta Tsutsui, Joachim Behar, Jack M. Moen, Yosuke Okamoto, Toni‐Rose Guiriba, Jie Liu, Rostislav Bychkov, Edward G. Lakatta

**Affiliations:** ^1^ Biomedical Engineering FacultyTechnion‐IITHaifaIsrael; ^2^ Laboratory of Cardiovascular ScienceBiomedical Research CenterIntramural Research ProgramNational Institute on AgingNIHBaltimoreMDUSA

**Keywords:** autonomic neural impulses, chaotic systems, heart rate variability, intrinsic heart rate, pacemaker cells, sinoatrial nodal

## Abstract

We aimed to determine how age‐associated changes in mechanisms extrinsic and intrinsic to pacemaker cells relate to basal beating interval variability (BIV) reduction *in vivo*. Beating intervals (BIs) were measured in aged (23–25 months) and adult (3–4 months) C57BL/6 male mice (i) via ECG *in vivo* during light anesthesia in the basal state, or in the presence of 0.5 mg mL^−1^ atropine + 1 mg mL^−1^ propranolol (*in vivo* intrinsic conditions), and (ii) via a surface electrogram, in intact isolated pacemaker tissue. BIV was quantified in both time and frequency domains using linear and nonlinear indices. Although the average basal BI did not significantly change with age under intrinsic conditions *in vivo* and in the intact isolated pacemaker tissue, the average BI was prolonged in advanced age. *In vivo* basal BIV indices were found to be reduced with age, but this reduction diminished in the intrinsic state. However, in pacemaker tissue BIV indices increased in advanced age vs. adults. In the isolated pacemaker tissue, the sensitivity of the average BI and BIV in response to autonomic receptor stimulation or activation of mechanisms intrinsic to pacemaker cells by broad‐spectrum phosphodiesterase inhibition declined in advanced age. Thus, changes in mechanisms intrinsic to pacemaker cells increase the average BIs and BIV in the mice of advanced age. Autonomic neural input to pacemaker tissue compensates for failure of molecular intrinsic mechanisms to preserve average BI. But this compensation reduces the BIV due to both the imbalance of autonomic neural input to the pacemaker cells and altered pacemaker cell responses to neural input.

## Introduction

It has been known since the 18th century that healthy heart beating intervals (BIs) are not strictly constant, but rather exhibit beat‐to‐beat variations, imparting complexity to the heart rhythm (Fye, 1995). Beating Interval Variability (BIV) reduction is a predictor of heart diseases and an increased mortality rate (reviewed in Yaniv *et al*. (2013b). Although the average basal BI remains constant with advancing age, the basal BIV is found to be reduced (Craft & Schwartz, 1995). In contrast to the preservation of the average basal BI, the average intrinsic BI, that is, in the absence of autonomic neural input, is found to be prolonged with advanced age (Jose & Collison, 1970). Whether and how the intrinsic BIV is altered in advanced age and the identities of mechanisms that underlie the changes in the BI–BIV relationship that accompany advancing age have not been well characterized.

Two main mechanisms regulate the average BI and BIV: (i) stimulation of extrinsic autonomic receptors on pacemaker cells (i.e. β‐adrenergic receptors (β‐AR) or cholinergic receptors (CR)) within the sinoatrial node (SAN) (O'Brien *et al*., 1986; Sloan *et al*., 2008) controlled by the balance between sympathetic and parasympathetic neural impulses to the heart and (ii) constitutive signaling intrinsic to pacemaker cell via Ca^2+^‐calmodulin adenylyl cyclase (AC) types 1 and 8 (Papaioannou *et al*., 2013; Ben‐Ari *et al*., 2014; Yaniv *et al*., 2014a,b), which, in the absence of autonomic receptor stimulation, drives many of the same cell mechanisms that are modulated by autonomic receptor stimulation (Mattick *et al*., 2007; Younes *et al*., 2008).

Both neural input to pacemaker cells and mechanisms intrinsic to pacemaker cells deteriorate with advancing age: (i) Age‐dependent remodeling of ion channels, connexins, internal Ca^2+^ mechanisms and a reduced spontaneous action potential (AP) rate have been documented in the isolated rat pacemaker cells (Jones *et al*., 2004; Tellez *et al*., 2006); (ii) an age‐associated reduction in Ca^2+^‐activated AC‐cAMP/protein kinase A (PKA) signaling and a prolonged Ca^2+^ transient have been documented in mice (Liu *et al*., 2014); (iii) in mammals, including humans, aging is associated with increased sympathetic neural impulses (reviewed in Lakatta (1993)) and reduced responses of the average BI to β‐AR receptor stimulation or CR stimulation (reviewed in Brodde & Leineweber (2004)) both *in vivo*, and specifically in pacemaker tissue *ex vivo* (Liu *et al*., 2014); (iv) a maximal heart rate reduction is related to age‐associated alteration of mechanisms both extrinsic to and intrinsic to pacemaker cells in mice and humans (Christou & Seals, 2008; Larson *et al*., 2013).

Although changes in both extrinsic and intrinsic signaling cascades have been implicated in the age‐associated preservation of average basal BI, whether mechanisms intrinsic to pacemaker cells are involved in age‐associated reduction in BIV *in vivo*, and how these intrinsic mechanisms impact age‐associated changes in BI–BIV relationship *in vivo* remains to be elucidated. We hypothesized that age‐associated changes in average BI and BIV result from the alteration in both intrinsic and neural input signaling. We analyzed BI dynamics in mice of varying ages: (i) *in vivo*, when the autonomic input to the sinoatrial node is intact; (ii) during autonomic denervation *in vivo*; and (iii) *ex vivo*, in the intact isolated SAN tissue (i.e. in which the autonomic neural input is absent). Our results confirm the hypothesis that deterioration of mechanisms both intrinsic to and extrinsic to pacemaker cells contributes to age‐associated changes in BI–BIV relationship *in vivo*.

## Results

### Intrinsic and neural input mechanisms differentially affect the average BI and BIV throughout the adult lifespan

**Figure 1:**
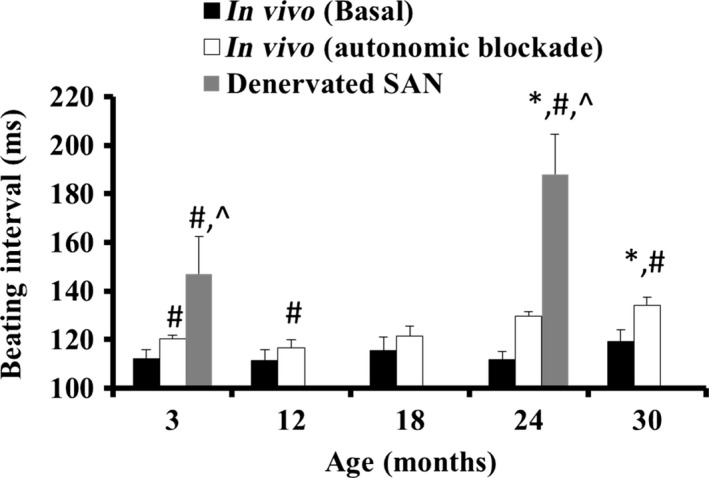
Age‐associated changes in beating rate in both *in vivo* and denervated SAN. *P < 0.05 vs. 3 months, ^#^ *P* < 0.05 vs. basal *in vivo* at the same age, ^*P* < 0.05 vs. intrinsic *in vivo* at the same age.

To determine whether and how autonomic neural input and mechanisms intrinsic to the SAN are involved in age‐associated preservation of the average BI and reduction in BIV, we quantified these (i) in the basal *in vivo* state; (ii) during an intrinsic autonomic denervation state *in vivo*; and (iii) *ex vivo*, in the intact isolated SAN tissue, in which the autonomic neural input is absent. The basal average BI *in vivo* did not significantly differ from 3 to 30 months (Fig. 1, Table S1, Supporting information). In contrast, the average intrinsic BI *in vivo* began to be prolonged at 18 months, but prolongation reached a statistical significance at 30 months (Table S2). In the isolated SAN, the average BI was already significantly prolonged at 24 compared to 3 months (Fig. 1). Given that 50% mortality rate occurs at 24 months, we defined 3‐month‐old mice as an adult population and 24‐month‐old mice as an aged population.

**Figure 2:**
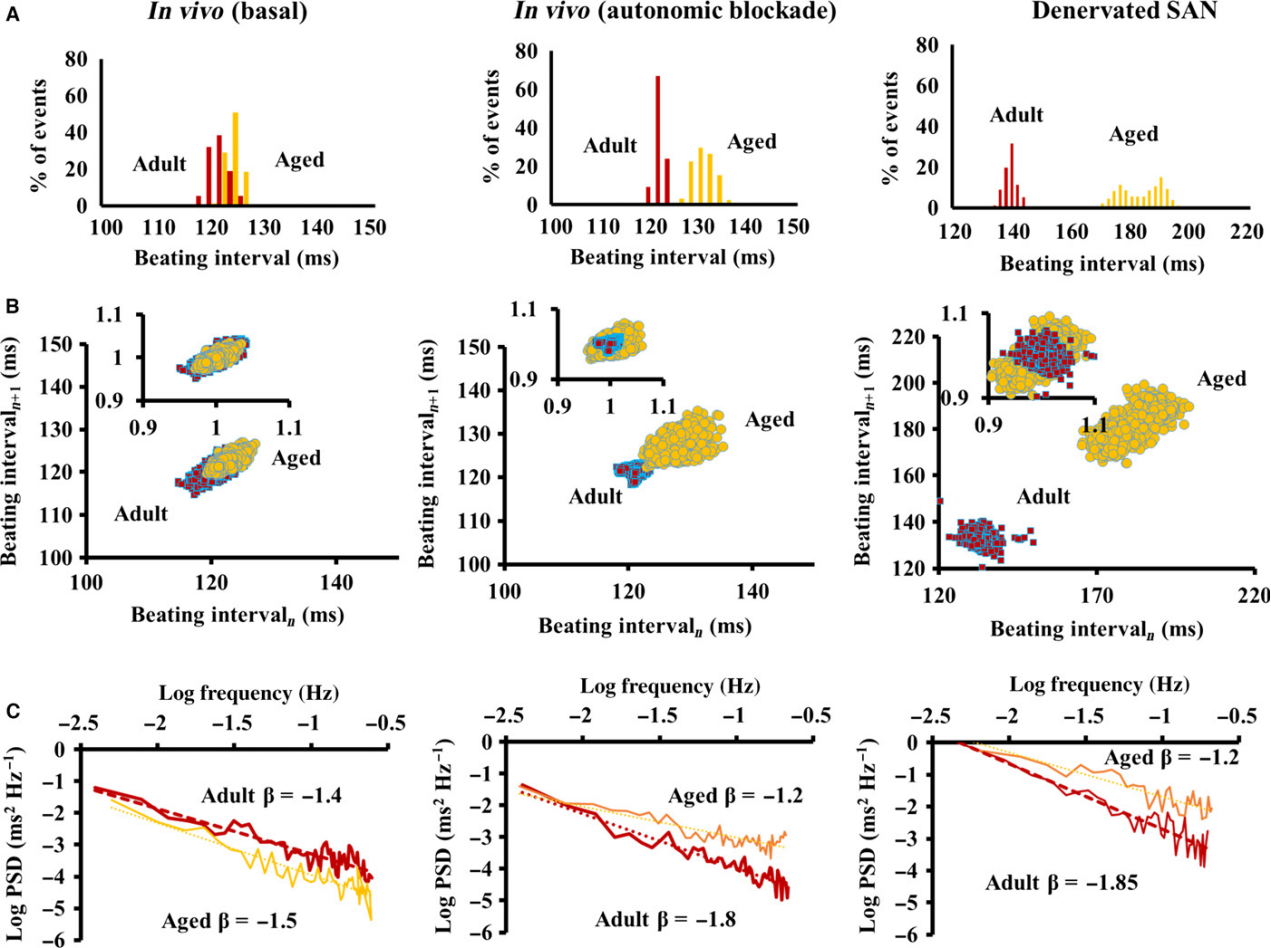
Beating interval distributions *in vivo* and in the isolated SAN tissue. Representative examples of (A) distributions of beating intervals, (B) Poincaré plots of the beating intervals (insets: normalized plots), and (C) power‐law behavior (log power spectrum density—PSD vs. log frequency) of beating intervals *in vivo*,* in vivo* following the autonomic blockade and in the isolated SAN *ex vivo*.

Figure 2A illustrates representative examples of BI histograms recorded *in vivo*,* in vivo* during autonomic denervation, and in the SAN tissue *ex vivo*. Autonomic blockade *in vivo* prolonged the average BI vs. that in the basal state *in vivo*, and variations of intrinsic BI were found to be reduced compared to those in the basal state. In the isolated SAN tissue, the average BI was also longer than the *in vivo* conditions and the variations of BI were greater.

Although the average basal BI *in vivo* did not significantly change with aging, the BIV was less than in the adults (Fig. 2A). The average intrinsic BI measured *in vivo* or in the isolated SAN tissue was longer in advanced age compared to adults with increased BIV in the isolated SAN tissue (Fig. 2A). Table S3 lists the average BIV time‐domain parameters in adult and aged mice (see online supplement for definitions), which in the basal state tended to be lower in advanced age compared to the adults (only the coefficient of variation (CV) was significant). This tendency reduced following autonomic denervation *in vivo* and in the isolated SAN tissue tended to be higher in advanced age (only CV reached significance in the isolated SAN). These trends for BIV alterations with age are illustrated in Poincaré plots, in which each beating cycle length is plotted against its predecessor to quantify the correlation between consecutive BIs (Fig. 2B). Similar to the trends in linear BIV indices, basal *in vivo* approximate entropy (ApEn, Table S3), which defines the degree of complexity among BIs, is found to be reduced in advanced age *in vivo*, but is increased in advanced age in intrinsic states *in vivo* and in the isolated SAN tissue.

Table S3 lists frequency‐domain parameters calculated in adult and aged mice. In basal *in vivo*, ratio of low frequency (LF) to total frequency (total) increased with aging, as the ratio of high frequency (HF) to total and ratio of LF to HF decreased. These basal *in vivo* age differences were abolished in the intrinsic state *in vivo* and in the isolated SAN tissue. Compared to both *in vivo* conditions, the ratio of very low frequency (VLF) to total in the isolated SAN tissue did not vary with age.

Power‐law analyses and detrended fluctuation analysis (DFA) quantify fractal‐like behavior embedded within BIs signaling. System variability is defined by its fractal scaling exponent, β, that is, the slope of a linear function relating log frequency to log of the power spectrum density within VLF domain. In the basal state *in vivo*, β did not significantly differ with age (Fig. 2C, Table S3). During the autonomic denervation *in vivo* or in the isolated SAN, however, β significantly increased beyond the basal value *in vivo* in the adults, but was found to be reduced in advanced age (Fig. 2C, Table S3). DFA characterizes the degree of correlation among time scales embedded within the heart BIs (Goldberger *et al*., 2000). DFA exhibited similar age‐associated trends as those identified by fractal analysis, but not all were statistically significant (Table S3).

To verify that the light general anesthesia did not affect the trend of our *in vivo* results, we performed additional BIV analysis in a subset of awake animals with implanted telemetry sensors (Data Sciences International, St Paul, MN). Similar BIV reduction under intrinsic vs. basal conditions in adult mice and no changes under intrinsic vs. basal conditions in aged mice were found in the awake animals (Table S4, Fig. S2). Note, however, that the time‐domain BIV indices tended to be higher in the awake than in those under light general anesthesia as documented before (Pham *et al*., 2009).

### Blend response of autonomic neural input receptor stimulation in advanced age

**Figure 3:**
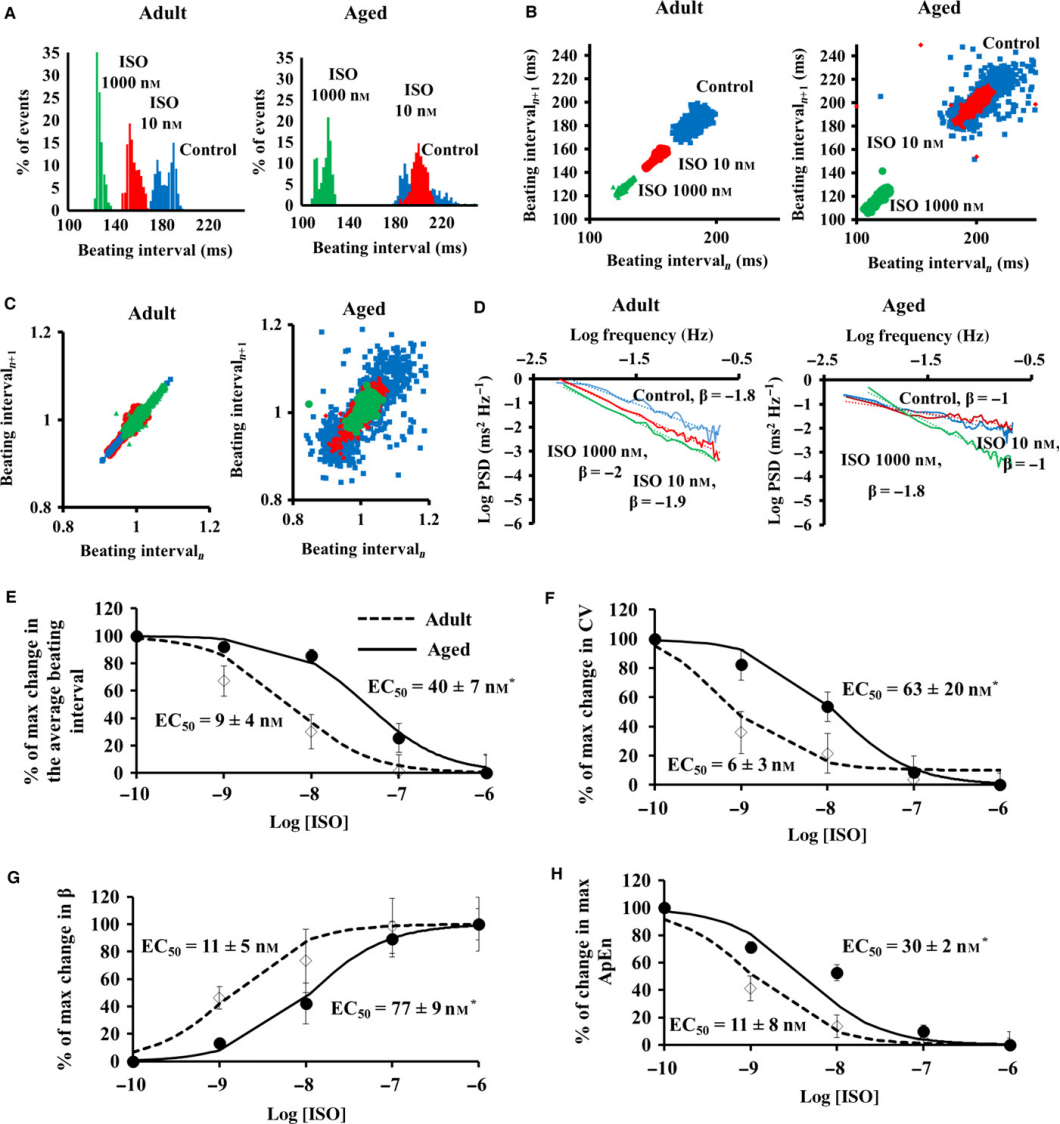
Beating interval dynamics in response to β adrenergic receptor stimulation by isoproterenol (ISO) in the isolated SAN *ex vivo*. Representative examples of (A) distribution of beating intervals, (B) regular and (C) normalized Poincaré plots of the beating intervals, and (D) power‐law behavior (log power spectrum density—PSD vs. log frequency) of beating interval response to β‐AR stimulation by ISO in the isolated SAN *ex vivo*. Percent of maximal changes in (E) beating rate, (F) coefficient of variance (CV), (G) fractal scaling exponent (β), and (H) approximate entropy in response to different degrees of β‐AR stimulation by isoproterenol (ISO) in the SAN tissue isolated from adult (*n* = 6) and aged (*n* = 6) mice.

To identify the extent to which the age‐associated changes in sensitivity of autonomic receptor stimuli contribute to the age‐associated preservation of BI and reduction in BIV, we employed a selective autonomic blockade or activation in the isolated SAN tissue. Representative examples of BI histogram and BIV indices in response to β‐AR stimulation are shown in Fig. 3. In response to the graded β‐AR stimulation (Fig. S3), a reduction in the average BI was paralleled by a decrease in BIV, illustrated by BI histogram (Fig. 3A) and in the Poincaré plots (Fig. 3B–C), and quantified by linear indices (Table S5), fractal‐like complexity slope (Table S5, Fig. 3D), and system entropy (Table S5). Compared to the adults, however, the sensitivity of average BI and BIV to β‐AR stimulation was found to be reduced in advanced age (Fig. 3E–H). Time controls for the changes in BI and BIV at both ages are listed in Table S6.

**Figure 4:**
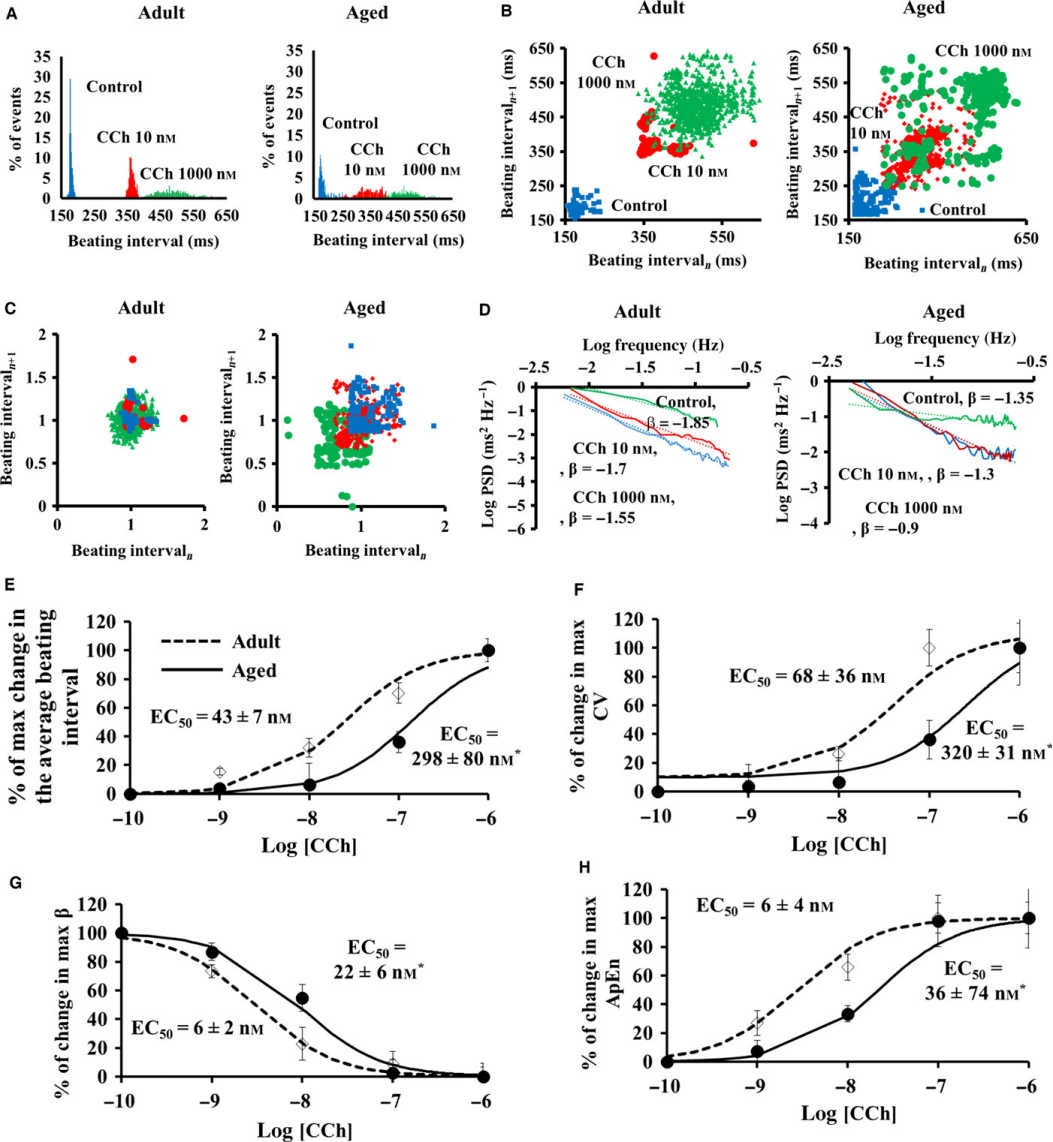
Beating interval dynamics in response to cholinergic receptor stimulation by carbachol (CCh) in the isolated SAN *ex vivo*. Representative examples of (A) distribution of beating intervals, (B) regular and (C) normalized Poincaré plots of the beating intervals, and (D) power‐law behavior (log power spectrum density—PSD vs. log frequency) of beating intervals response to CR stimulation by carbachol CCh in the isolated SAN *ex vivo*. Percent of maximal changes in (E) beating rate, (F) coefficient of variance (CV), (G) fractal scaling exponent (β), and (H) approximate entropy in response to different degrees of CR stimulation by carbachol (CCh) in the SAN tissue isolated from adult (*n* = 6) and aged (*n* = 6) mice.

Representative examples of BI histograms and BIV indices of responses to CR stimulation in the isolated SAN tissue are illustrated in Fig. 4. CR, in contrast to β‐AR stimulation, increased the average BI and BIV, illustrated by BI histogram (Fig. 4A) and in the Poincaré plots (Fig. 4B–C), and quantified by linear indices (Table S7), fractal‐like complexity slope (Table S7, Fig. 4D), and system entropy (Table S7). Note that compared to adults, the sensitivity to CR stimulation of average BI and BIV was found to be reduced in advanced age (Fig. 4E–H).

### Deterioration of mechanisms intrinsic to pacemaker cells in advanced age

**Figure 5:**
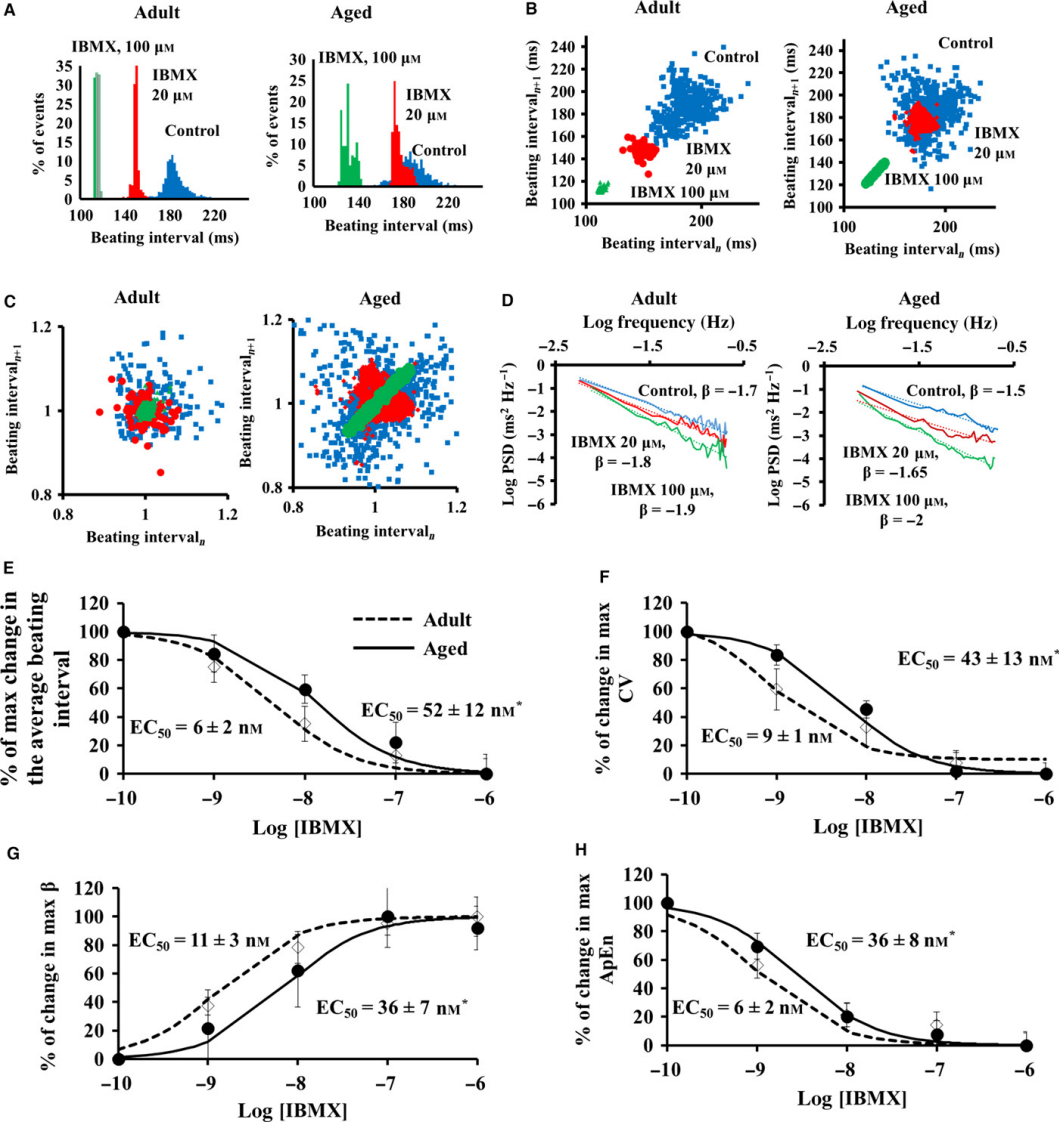
Beating interval dynamics in response to PDE inhibition by 3′‐isobutylmethylxanthine (IBMX) in the isolated SAN *ex vivo*. Representative examples of (A) distribution of beating intervals, (B) regular and (C) normalized Poincaré plots of the beating intervals, and (D) power‐law behavior (log power spectrum density—PSD vs. log frequency) of beating interval response to PDE inhibition by 3′‐isobutylmethylxanthine (IBMX) in isolated SAN *ex vivo*. Percent of maximal changes in (E) beating rate, (F) coefficient of variance (CV), (G) fractal scaling exponent (β), and (H) approximate entropy in response to different degrees of PDE inhibition by IBMX in the SAN tissue isolated from adult (*n* = 6) and aged (*n* = 6) mice.

Reduced sensitivity to autonomic receptor agonists may, in part, be attributable to the mechanisms affecting the cAMP‐PKA signaling distal to surface membrane receptors. To test whether distal mechanisms to β‐AR lead to the reduced sensitivity, we employed a broad‐spectrum phosphodiesterase (PDE) inhibitor (IBMX) to increase the cAMP/PKA signaling in the isolated SAN tissue (Vinogradova *et al*., 2008; Liu *et al*., 2014). Representative examples and the average data of the average BI and BIV responses to PDE inhibition are shown in Fig. 5. Note that the responses to PDE inhibition mimic those to β‐AR stimulation: a reduction in average BI accompanied by a reduction in BIV, illustrated by BI histogram (Fig. 5A) and in the Poincaré plots (Fig. 5B–C), and quantified by linear indices (Table S8), fractal‐like complexity slope (Table S8, Fig. 5D), and system entropy (Table S8). Similar to the response to β‐AR stimulation, the sensitivity of BI and BIV to PDE inhibition was found to be reduced in advanced age compared to adults (Fig. 5E–H).

## Discussion

Our major novel finding is that in addition to the changes in autonomic neural signaling with advanced age that were documented before (Beckers *et al*., 2006; Corino *et al*., 2007; Albinet *et al*., 2010), changes in mechanisms intrinsic to pacemaker cells regulate the age‐associated changes in average BI and BIV *in vivo*.

### Changes in intrinsic vs. neural receptor stimulation mechanisms in advanced age

**Figure 6:**
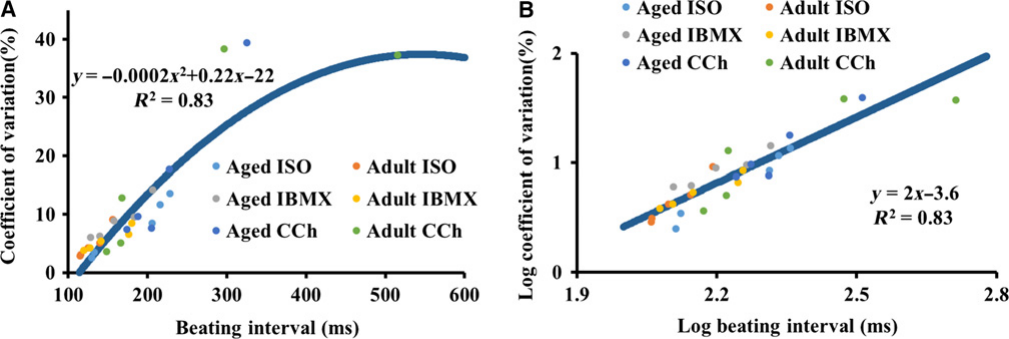
The relationship between beating intervals and coefficients of variation in the isolated SAN in (A) linear and in (B) log–log scale.

Reduced sensitivity to either neural impulse input or mechanisms intrinsic to pacemaker cells in advanced age may contribute to age‐associated changes in average BI and BIV *in vivo*: The deterioration of mechanisms intrinsic to the SAN leads to an increased average BI and BIV, and the extrinsic mechanisms (altered sympathetic to parasympathetic neural input) compensate for deteriorated intrinsic mechanisms to preserve the average BI. But this compensation is associated with BIV reduction (Fig. 2) due to both imbalance between autonomic compensatory neural input to the pacemaker cells, and altered pacemaker cell responses to neural input (Figs 3, 4, 5). Because the BI–BIV relationship at different ages (Fig. 6) is nonlinear by nature, as discovered previously in the isolated pacemaker cells (Zaza & Lombardi, 2001), one can assume that compensatory autonomic neural input to the SAN (increased sympathetic and/or increased parasympathetic) only returns the prolonged BI *in vivo* observed in advanced age to its adult value (Fig. 2), but does not return the BIV to its adult value.

Our experiments lead to a number of novel conclusions that relate age‐associated changes in neural receptor stimulation and intrinsic mechanisms to pacemaker cells: (i) As documented previously (Margneffe *et al*., 1988), because the average basal BI *in vivo* does not change throughout the lifespan (i.e. from 3 to 30 months), but the intrinsic average BI begins to decline at 18 months (Fig. 1), the ratio of sympathetic to parasympathetic neural input must increase in advanced age. An increase in sympathetic activity is known to reduce both BI and BIV, while a reduced parasympathetic activity leads to similar trends (for review, see Yaniv *et al*. (2013b)). Age‐associated trends that have been demonstrated previously (for review, see Yaniv *et al*. (2013b)) strengthen our conclusion regarding the age‐associated increase in sympathetic activity: An increase in sympathetic activity steepens the fractal‐like slope in mammals and increases the LF‐to‐total power ratio (Table S3); increase in sympathetic activity increases the ratio of LF to HF (Table S3). That these trends in the frequency domain are abolished in intrinsic conditions implies the role of neural system in BIV. (ii) The present study documents, for the first time in the isolated SAN, that the sensitivity of BIV responses to either muscarinic or adrenergic receptor activation is also found to be reduced in advanced age (Figs 3 and 4). Thus, the sympathetic‐to‐parasympathetic ratio must increase in advanced age in order to preserve the average basal BI due to reduced intrinsic mechanisms (Figs 3 and 4), the SAN in advanced age is ‘stuck in low gear’ with respect to the BI and BIV responses to neurotransmitters and higher sympathetic to parasympathetic neurotransmissions are required. Indeed, plasma levels of norepinephrine and epinephrine are higher in advanced aged humans (Fleg *et al*., 1985). The reduced sensitivity to autonomic stimulation may be due to a reduced number of receptors or desensitization of the receptors of pacemaker cells residing in the SAN. Future experiments in pacemaker cells are needed to solve this enigma. (iii) An additional novel finding of the present study is that the sensitivity of BIV to PDE inhibition also becomes reduced with age (Fig. 5). Because intrinsic mechanisms involving cAMP/PKA signaling drive intrinsic pacemaker clock mechanisms in the absence of external stimulation (reviewed in Yaniv *et al*. (2015)), the effect of PDE inhibition not only further supports the conclusion that changes in intrinsic mechanisms are involved in changes in BIV *in vivo*, but may also suggest an altered AC‐cAMP/PKA signaling in advanced age. Mechanisms intrinsic to the SAN cells have been documented in advanced age: (a) down‐regulation of HCN channels in rat SAN (Huang *et al*., 2007); (b) reduced gene expression of RyR and K_V_1.5 and increased gene expression of Na_v_1.5, Na_v_β1, and Ca_v_1.2 in rat SAN (Tellez *et al*., 2006); (c) an increased PLB‐to‐SERCA ratio and a reduced NCX1‐to‐RyR2 ratio in mouse SAN (Liu *et al*., 2014). The combined effects of age‐associated Ca^2+^‐activated cAMP/PKA signaling (Liu *et al*., 2014) are likely involved in the age‐associated increase in the average intrinsic BI and BIV. Because cAMP/PKA signaling matches ATP supply to demand in pacemaker cells (Yaniv *et al*., 2013c), age‐associated changes in Ca^2+^‐activated cAMP/PKA signaling can lead to an energetic imbalance and may also be implicated in intrinsic BI prolongation (Yaniv *et al*., 2013a).

### BI and BIV of humans vs. mice in advanced age

The major advantage of using mice as an aged model is their short lifespan (50% mortality rate around 24 months). Similarities between age‐associated trends documented here in mice and those documented in humans strengthen the reliability of the aging mice model: (i) In healthy subjects, basal average BI does not change with age, but the average intrinsic BI prolongs with age when compared to adults (Jose & Collison, 1970); (ii) basal HRV indices in humans *in vivo* decrease (Goldberger *et al*., 2002; Beckers *et al*., 2006); specifically, β and ApEn are found to be reduced in advanced age (Pikkujamsa *et al*., 1999); (iii) an age‐associated reduction in the sensitivity of the average BI to ISO also occurs in humans: The EC_50_ values for ISO for older subjects were nearly twice those in younger subjects (White *et al*., 1994). Note, however, that the intrinsic average BI in humans becomes reduced during double autonomic blockade (Tan *et al*., 2009) compared to average BI prolongation in mice. This disparity is due to a higher ratio of parasympathetic to sympathetic activity in humans than in mice. In both models however, the parasympathetic‐to‐sympathetic ratio decreases in advanced age.

In response to β‐AR stimulation or PDE inhibition, the changes in average BI and BIV of the isolated SAN tissue of advanced age are restored to the basal levels in adults (Fig. S3). Partially, because β‐AR stimulation of the isolated pacemaker cells can restore pacemaker cell BIV characteristics to that of the intact SAN tissue (Yaniv *et al*., 2014a), and that increasing β‐AR stimulation in the isolated pacemaker tissue shift the leading pacemaker site (Lang *et al*., 2011). Therefore, one may conclude that restoring the activities of intrinsic and extrinsic mechanisms to pacemaker cells may rescue the old heart. Future experiments on the isolated pacemaker cells and the SAN tissue are necessary to prove these predictions.

### Clinical applications

As discussed above, BIV reduction is not only associated with aging, but it is a predictor of heart diseases and an increased mortality rate. Recently, more evidence has emerged to indicate that in addition to the changes in autonomic neurotransmitters, a reduction in intrinsic pacemaker cell function is implicated in altered HRV associated with cardiac diseases (reviewed in Yaniv *et al*. (2013b). One can hypothesize, therefore, that a similar trend of BIV can occur in cardiac disease as in aging: Changes in the intrinsic pacemaker mechanisms lead to a prolongation in the average BI and an increase in BIV, and as a response, overcompensation by the autonomic neural input maintains the average BI, but leads to reduced BIV. Early diagnosis of and therapies for cardiac disease and identification of their association with pacemaker cell dysfunction can prevent overcompensation by the autonomic neural input and may reduce mortality.

### Limitations

We found similar trends of intrinsic BI and BIV *in vivo* and in the isolated tissue in the present study (Fig. 2). However, factors other than autonomic neural input, that is, sympathetic and parasympathetic nerve stimulation, also modulate BI and BIV *in vivo* and are likely present during autonomic blockade (for review, see Yaniv & Lakatta (2015). The other factors lead to changes in the patterns of intrinsic BI and BIV observed *in vivo* and in the isolated completed SAN tissue. Note that the similar trends of intrinsic BI and BIV *in vivo* and in the isolated tissue and the ability to restore BI and BIV of aged isolated SAN tissues to the level of the young SAN tissues in response to ISO application imply that there are no age‐dependent technical limitations to our isolation technique.

### Conclusions

In advanced age, both reduced sensitivity of intrinsic pacemaker mechanisms to cAMP/PKA signaling and reduced sensitivity to autonomic neural input lead to changes in BIV *in vivo*. Reduced sensitivity of intrinsic pacemaker mechanisms to cAMP/PKA signaling leads to both increased BI and BIV. Changes in autonomic receptor stimulation compensate for the prolonged intrinsic pacemaker cell clock period, shifting the average BI period *in vivo* to a lower value, while reducing its complexity. Therefore, only a complete understanding of changes in intrinsic and extrinsic pacemaker mechanisms that accompany advanced age can lead to treatments that will improve lifespan and quality.

## Experimental procedures

### Heart rate measurements *in vivo*

All animal studies were performed in accordance with the Guide for the Care and Use of Laboratory Animals published by the National Institutes of Health (NIH Publication no. 85‐23, revised 1996). Experimental protocols were approved by the Animal Care and Use Committee of the National Institutes of Health (protocol #441LCS2013). The ECG was recorded at 3‐ to 4‐month‐old (*n* = 17), at 12‐ to 13‐month‐old (*n* = 6), at 18‐ to 20‐month‐old (*n* = 9), at 24‐ to 25‐month‐old (*n* = 21), and at 30‐ to 32‐month‐old (*n* = 9) C57/BL6 mice, which were sedated with isoflurane (2% in oxygen) via a nosecone and kept at 37 °C using a heating pad and lamp. Standard ECG electrodes were placed on the limbs and lead II was recorded continuously, using a Power Lab system (AD instruments, Sydney, Australia) at a sampling rate of 1 KHz (Fig. S1). After a 20‐min stabilization period followed by 10‐min baseline ECG recording, atropine (0.5 mg kg^−1^) and propranolol (1 mg kg^−1^) diluted in saline (6.6 mL kg^−1^) were given i.p. and the ECG was recorded for another 40 min.

### SAN tissue isolation and beating rate measurements

Hearts were quickly excised and placed into HEPES‐buffered solution (36 ± 0.5°C) of the following composition (in mm): 137 NaCl, 4.9 KCl, 1 MgCl_2_, 20 HEPES, 1.2 NaH_2_PO_4_, 5 NaHCO_3_ 1.8 CaCl_2_, and 16.6 glucose, titrated to pH 7.2 with NaOH and bubbled with O_2_. The right and left atria were dissected together with the SAN region. The sinoatrial node strip, defined as the region bordered by its anatomic landmarks (the crista terminalis, the interatrial septum, and the superior and inferior vena cavae), was dissected from 3‐ to 4‐month‐old (*n* = 21) and 24‐ to 25‐month‐old (*n* = 21) C57/BL6 mice. The SAN preparation was fixed in a heated bath (36 ± 0.5 °C) and superfused with the HEPES‐buffered solution at a rate of 8 mL min^−1^. Following 20 min of stabilization, an insulated custom‐made stainless steel electrode with a tip of 0.15 mm diameter was placed in the center of the SAN to record the extracellular electrogram (Fig. S1) using a Neurolog system NL900D (Digitimer, Hertfordshire, UK). Extracellular electrograms were recorded for 10 min under control conditions and 10 min after each drug application. BIs were analyzed by a custom‐made matlab program (MathWorks). See online supplement for the description of methods to analyze BIV.

### Drugs

Isoproterenol, carbachol, 3′‐isobutylmethylxanthine (IBMX), and isoflurane were purchased from Sigma.

### Statistical analyses

All data are presented as mean ± SD. A mixed‐effects model was used to determine the age, drug, and interaction effects for repeated‐measures data. For further details, see the Supporting information.

## Funding

This research was partially supported by the Intramural Research Program of the NIH, National Institute on Aging, sponsored in part by a Glenn Foundation Cube Award for research on mechanisms of biologic aging (E.G.L)., by Technion V.P.R Fund‐Mallat Family Research Fund (Y.Y.), Ministry of Science (Y.Y), and by NSFC‐ISF joint research program, No. 398/14 (Y.Y).

## Conflict of interest

None declared.

## Author contributions

EGL YY conceived and designed the experiments. YY IA, KT, and JMM performed the experiments. YY AEL JB analyzed the data. YO, TRG, JL, and RB contributed reagents/materials/analysis tools. EGL YY wrote the manuscript.

## Supporting information


**Fig. S1** ECG recording *in vivo* and electrogram recording in isolated SAN.
**Fig. S2** Age‐associated changes in (A) beating interval, (B) coefficient of variance (CV) and (C) low frequency (LF) to high frequency (HF) ratio in awake mice *in vivo* (*n* = 3) and during autonomic blockade (DB).
**Fig. S3** Dose–response of beating rate, coefficient of variance (CV) and approximate entropy (ApEn) in response to different degrees of (A) β‐AR stimulation by isoproterenol (ISO), (B) CR stimulation by carbachol (CCh) or (C) PDE inhibition by 3′‐isobutylmethylxanthine (IBMX) in the isolated SAN tissue from adult (*n* = 6) and aged (*n* = 6) mice for each drug intervention.
**Table S1** Measures of basal beating interval dynamics *in vivo*.
**Table S2** Measures of beating interval dynamics during autonomic neural input blockade (intrinsic conditions) and time controls *in vivo*.
**Table S3** Measures of beating interval dynamics *in vivo* and in isolated tissue.
**Table S4** Measures of beating interval dynamics *in vivo* in awake mice.
**Table S5** Measures of beating interval dynamics in isolated SAN tissue in response to β‐adrenergic receptor stimulation.
**Table S6** Time controls for measures of beating interval dynamics in isolated SAN tissue.
**Table S7** Measures of beating interval dynamics in isolated SAN tissue in response to muscarinic receptor stimulation.
**Table S8** Measures of beating interval dynamics in isolated SAN tissue in response to phosphodiesterase inhibition.
**Data S1** Extended methods.
