# Supplementary material for: Deterioration of autonomic neuronal receptor signaling and mechanisms intrinsic to heart pacemaker cells contribute to age‐associated alterations in heart rate variability in vivo
Source: Aging Cell. 2016 May 10;15(4):716–24. doi: 10.1111/acel.12483 (PMC4933656; doi:10.1111/acel.12483)
Supplement: Supplementary file 1 — Fig. S1 ECG recording in vivo and electrogram recording in isolated SAN. Fig. S2 Age‐associated changes in (A) beating interval, (B) coefficient of variance (CV) and (C) low frequency (LF) to high frequency (HF) ratio in awake mice in vivo (n = 3) and during autonomic blockade (DB). Fig. S3 Dose–response of beating rate, coefficient of variance (CV) and approximate entropy (ApEn) in response to different degrees of (A) β‐AR stimulation by isoproterenol (ISO), (B) CR stimulation by carbachol (CCh) or (C) PDE inhibition by 3′‐isobutylmethylxanthine (IBMX) in the isolated SAN tissue from adult (n = 6) and aged (n = 6) mice for each drug intervention. Table S1 Measures of basal beating interval dynamics in vivo. Table S2 Measures of beating interval dynamics during autonomic neural input blockade (intrinsic conditions) and time controls in vivo. Table S3 Measures of beating interval dynamics in vivo and in isolated tissue. Table S4 Measures of beating interval dynamics in vivo in awake mice. Table S5 Measures of beating interval dynamics in isolated SAN tissue in response to β‐adrenergic receptor stimulation. Table S6 Time controls for measures of beating interval dynamics in isolated SAN tissue. Table S7 Measures of beating interval dynamics in isolated SAN tissue in response to muscarinic receptor stimulation. Table S8 Measures of beating interval dynamics in isolated SAN tissue in response to phosphodiesterase inhibition. Data S1 Extended methods. [file ACEL-15-716-s001.doc]

**On-line supplement**

**Deterioration of autonomic neuronal receptor signaling and mechanisms intrinsic to heart pacemaker cells contribute to age-associated alterations in heart rate variability in vivo**

**Yael Yaniv^1*^, Ismayil Ahmet^2^, Kenta Tsutsui^2^, Joachim Behar^1^, Jack M. Moen^2^, Yosuke Okamoto^2^, Toni-Rose Guiriba^2^, Jie Liu^2^, Rostislav Bychkov^2^, and Edward G. Lakatta^2*^**

^1^Biomedical Engineering Faculty, Technion-IIT, Haifa, Israel. ^2^Laboratory of Cardiovascular Science, Biomedical Research Center, Intramural Research Program, National Institute on Aging, NIH, Baltimore, Maryland, USA.

***Corresponding authors:**

Yael Yaniv, PhD

Email: [yaely@bm.technion.ac.il](mailto:yaely@bm.technion.ac.il)

Telephone: +972-4-8294124

Fax: +972-4-8294599

Edward Lakatta, MD

Email: [lakattae@grc.nia.nih.gov](mailto:lakattae@grc.nia.nih.gov)

Telephone: 1-410-558-8202

Fax: 1-410-558-8150

**Extended methods**

**Linear time-domain methods for heart rate or beating rate variability**

All measurements were performed after the beating interval in each of the three conditions reached a steady state and at least 2048 beats were recorded.

***SDNN***

Standard deviation of all beating interval lengths (BIL) .

$$SDNN=\sqrt{\frac{1}{N}\sum_{i=1}^{N} (BIL_{i}-\mathop{BIL)}\limits^{-} }$$

$\mathop{BIL}\limits^{-}$is the arithmetic mean of the values of BIL.

***RMSDD***

Root mean square of successive BIL .

$$RMSSD=\sqrt{\frac{1}{N-1}\sum_{i=1}^{N-1} (BIL_{i+1}-BIL_{i})^{2}}$$

where N is the total number of beating intervals.

***CV***

Coefficient of variation is a normalized measure of dispersion of a probability distribution.

$$CV=\frac{SDNN}{\mathop{BIL}\limits^{\_} }$$

***pNN50***

Percentage of adjacent beating intervals differing by more than 50 ms .

$$pNN50=\frac{\sum_{i=1}^{N} \{\left|BIL_{i+1}-BIL_{i}>50ms\}\cdot100\right|}{N}$$

where N is the total number of BI.

**Frequency-domain methods for heart rate or beating rate variability**

Two raw frequency-domain statistics were extracted from each spectral density (2048 beats under in vivo conditions and in the intact SAN); the estimated power in each of three bands was calculated: very-low frequency (VLF), low frequency (LF), and high frequency (HF), the total power (Total) over all three bands, and LF to HF ratio. Due to different average beating rates in different fundamental states (i.e., in vivo and in isolated SAN) two different frequency regimes were defined as shown previously in rabbits : the VLF embodied frequency below 4% of the average beating rate, the LF domain included frequencies between VLF and 15% of the average beating rate, and the HF embodied frequencies between LF and 40% of the average beating rate. Note that these frequency domains are similar to those defined in humans . In vivo, VLF was below 0.26 Hz, LF was between 0.26 to 1 Hz and HF was between 1 to 3.25 Hz. In the isolated intact SAN VLF was below 0.2 Hz, LF was between 0.2 to 0.8 Hz and HF was between 0.8 and 2.2 Hz.

**Poincaré plot**

The Poincaré plot visualizes each beat interval as a function of the preceding interval to find correlation between them for both in vivo and isolated tissue levels.

**Power-Law analysis**

Fourier analysis was performed on the heart-beat intervals (see above). A robust line-fitting algorithm of log power spectrum density versus log frequency at VLF regime (defined separately for in vivo and isolated tissue) was applied. The slope of the line is the fractal scale exponent, β. A power law was assumed to be present if R^2^>0.7 .

**Detrended Fluctuation Analysis**

Detrended fluctuation analysis (DFA) quantifies the degree of complexity embedded within the heart beat intervals in the time domain . The root mean square fluctuations of the integrated and detrended data time series (F(n)) were calculated in two windows as described before . A robust line-fitting algorithm of log fluctuation versus log window size was applied to the short and long-term DFA windows. These bi-fractal slopes are described by short- and long-term exponents, α_1_ and α_2_, respectively.

**Approximate entropy (ApEn)**

Approximate entropy, a measure quantifying the regulatory of the time series, was calculated from the average values of segments encompassing 2048 beats with fixed input variables of m=2 and r=20% as previously described .

**Statistical analyses**

All data are presented as mean±SD. To test the effect of different ages at different functional levels (in vivo, autonomic denervation in vivo and intact SAN), a linear mixed-effects model was employed to determine whether the three functional states differed from each other at various ages and within each functional level with respect to the measured variables. The Sidak multiple comparison method was employed for post-hoc comparisons. For the repeated measures data (pacemaker tissue), a linear mixed-effects model was used to determine age, drug, and interaction effects. When the interaction was significant, we applied post-hoc Bonferroni approach restricted to the comparisons of interest, i.e. we multiplied the appropriate p-values by the number of comparisons being made. In particular, we compared the various drug effects within each age group and the various ages within each drug. All statistics were performed using SAS p.2 (SAS Institute Inc, NC). P<0.05 was considered as significant.

**References**

(1996a). Heart rate variability. Standards of measurement, physiological interpretation, and clinical use. Task Force of the European Society of Cardiology and the North American Society of Pacing and Electrophysiology. *Eur Heart J*. **17**, 354-381.

(1996b). Heart rate variability: standards of measurement, physiological interpretation and clinical use. Task Force of the European Society of Cardiology and the North American Society of Pacing and Electrophysiology. *Circulation*. **93**, 1043-1065.

Goldberger AL, Amaral LA, Glass L, Hausdorff JM, Ivanov PC, Mark RG, Mietus JE, Moody GB, Peng CK, Stanley HE (2000). PhysioBank, PhysioToolkit, and PhysioNet: components of a new research resource for complex physiologic signals. *Circulation*. **101**, E215-220.

Kobayashi M, Musha T (1982). 1/f fluctuation of heartbeat period. *IEEE transactions on bio-medical engineering*. **29**, 456-457.

Pincus SM, Goldberger AL (1994). Physiological time-series analysis: what does regularity quantify? *Am J Physiol*. **266**, H1643-1656.

Yaniv Y, Ahmet I, Liu J, Lyashkov AE, Guiriba TR, Okamoto Y, Ziman BD, Lakatta EG (2014). Synchronization of sinoatrial node pacemaker cell clocks and its autonomic modulation impart complexity to heart beating intervals. *Heart Rhythm*. **11**, 1210-1219.

| **Basal**  **parameter** | **3 months** | **12 months** | **18 months** | **24 months** | **30 months** |
| --- | --- | --- | --- | --- | --- |
| **Time-domain indices** | | | | | |
| **Average beating interval (ms)** | 112±4 | 111±5 | 116±6 | 115±4 | 119±5 |
| **SDNN (ms)** | 2.1±0.5 | 2±1 | 1.6±0.5 | 1.5±0.1 | 2.1±0.8 |
| **RMSSD (ms)** | 3.2±1 | 3.4±0.5 | 2.6±0.9 | 2.6±0.7 | 2.6±1 |
| **CV (%)** | 2±0.5 | 1.6±0.6 | 1.4±0.5 | 1.3±0.3^*^ | 1.7±0.7 |
| **pNN50 (%)** | 1±0.5 | 1±0.5 | 1.3±0.2 | 0±0^*^ | 0.1±0.1^*^ |
| **ApEn** | 2±0.2 | 1.9±0.2 | 1±0.1 | 1.8±0.1 | 1.8±0.1 |
| **Frequency indices** | | | | | |
| **VLF/Total (%)** | 21±3 | 32±9 | 18±5 | 15±3 | 22±5 |
| **LF/Total (%)** | 12±3 | 10±3 | 17±3 | 36±8^*^ | 23±6 |
| **HF/Total (%)** | 67±40 | 58±10 | 65±7 | 49±8^*^ | 55±10 |
| **LF/HF** | 69±4 | 54±10 | 67±7 | 49±8^*^ | 61±8 |
| **Non-linear indices** | | | | | |
| **β** | -1.3±0.2 | -1.3±0.3 | -1.5±0.1 | -1.5±0.3 | -1.7±0.2 |
| **α_1_** | 0.4±0.1 | 0.5±0.1 | 0.4±0.1 | 0.8±0.1^*^ | 0.6±0.2 |
| **α_2_** | 0.8±0.1 | 1±0.1 | 0.5±0.1 | 0.8±0.1 | 0.7±0.1 |

**Table S1:** Measures of **basal** beating interval dynamics in vivo. SDNN, standard deviation of the inter beat interval; RMSSD, root mean square of the successive beat interval differences; CV, coefficient of variation of beat interval; pNN50, percentage of adjoining beating intervals differing by more than 50 ms; VLF, very-low frequency power; LF, low frequency power; HF, high frequency power; Total, total spectrum power; ApEn, approximate entropy; β, slope of the power-law relationship; α_1_ short-term scaling exponent; and α_2_ long-term scaling exponent. Beating intervals were recorded at 3-4 months (n=17), at 12-13 months (n=6), at 18-20 months (n=9), at 23-25 months (n=21), and at 30-32 months (n=9) C57/BL6 mice. *p<0.05 vs. 3 months.

| **Intrinsic**  **parameter** | **3 months** | **12 months** | **18 months** | **24 months** | **30 months** | **Time- control**  **(3 months)** | **Time- control (24 months)** |
| --- | --- | --- | --- | --- | --- | --- | --- |
| **Time-domain indices** | | | | | | | |
| **Average beating interval (ms)** | 120±2^#^ | 117±3^#^ | 121±4 | 126±2 | 134±3^#,*^ | 119±5 | 127±5 |
| **SDNN (ms)** | 0.8±0.1^#^ | 0.8±0.1^#^ | 1±0.2 | 1.6±0.4 | 1.9±0.3^*^ | 1.0±0.2 | 1.5±0.1 |
| **RMSSD (ms)** | 1.1±0.2^#^ | 1.3±0.5^#^ | 1.3±0.3 | 1.4±0.3 | 3±1.2^*^ | 1.1±0.5 | 1.7±0.2 |
| **CV (%)** | 0.7±0.1^#^ | 0.7±0.1^#^ | 0.8±0.2 | 1.3±0.3 | 1.4±0.2^*^ | 1±0.2 | 1.4±0.1 |
| **pNN50 (%)** | 0.1±0.1 | 0.1±0.1 | 0.1±0.1 | 0.1±0.1 | 0.2±0.1 | 0.1±0.1 | 0.1±0.1 |
| **ApEn** | 1.3±0.2^#^ | 1.5±0.1^#^ | 1.2±0.1^#^ | 1.5±0.2^#^ | 1.8±0.1^#,*^ | 1.4±0.1 | 1.6±0.1 |
| **Frequency indices** | | | | | | | |
| **VLF/Total (%)** | 13±2^#^ | 18±6^#^ | 11±3 | 13±4 | 5±2^#^ | 18±4 | 16±2 |
| **LF/Total (%)** | 20±6 | 13±3 | 13±3 | 29±7 | 16±4 | 20±6 | 24±7 |
| **HF/Total (%)** | 67±9 | 69±9^#^ | 76±6 | 58±6 | 79±5^#^ | 62±5 | 60±5 |
| **LF/HF** | 67±9 | 72±9^#^ | 79±5 | 50±8 | 82±5^#^ | 56±9 | 70±25 |
| **Non-linear indices** | | | | | | | |
| **β** | -1.6±0.1 | -1.5±0.1 | -1±0.2^#,*^ | -1.2±0.1^#,*^ | -1.5±0.1 | -1.7±0.2 | -1.2±0.2^*^ |
| **α_1_** | 0.3±0.1 | 0.4±0.1 | 0.4±0.1 | 0.8±0.2^*^ | 0.4±0.2 | 0.4±0.2 | 0.7±0.2^*^ |
| **α_2_** | 0.5±0.1^#^ | 0.6±0.1 | 0.5±0.1 | 0.7±0.1^#^ | 0.5±0.1 | 0.6±0.1 | 0.6±0.2 |

**Table S2:** Measures of beating interval dynamics during autonomic neural input blockade (intrinsic conditions) and time controls in vivo. SDNN, standard deviation of the inter beat interval; RMSSD, root mean square of the successive beat interval differences; CV, coefficient of variation of beat interval; pNN50, percentage of adjoining beating intervals differing by more than 50 ms; VLF, very-low frequency power; LF, low frequency power; HF, high frequency power; Total, total spectrum power; ApEn, approximate entropy; β, slope of the power-law relationship; α_1_ short-term scaling exponent; and α_2_ long-term scaling exponent. Beating intervals were recorded at 3-4 months (n=17), at 12-13 months (n=6), at 18-20 months (n=9), at 24-25 months (n=21), and at 30-32 months (n=9) C57/BL6 mice. Time-controls (injection of saline) were correlated with the intrinsic measurements time. *p<0.05 vs. 3 months, ^#^p<0.05 vs. basal in vivo at the same age.

| **Parameter** | **Adult**  **Basal**  **In vivo** | **Aged**  **Basal**  **In vivo** | **Adult**  **Intrinsic**  **In vivo** | **Aged**  **Intrinsic**  **In vivo** | **Adult isolated tissue** | **Aged isolated tissue** |
| --- | --- | --- | --- | --- | --- | --- |
| **Time-domain indices** | | | | | | |
| **Beating rate (ms)** | 112±4 | 115±4 | 120±2 | 126±2 | 156±9^*,#^ | 214±10*,#,^ |
| **SDNN (ms)** | 2.1±0.5 | 1.5±0.1 | 0.8±0.1^*^ | 1.6±0.4 | 14±2^*,#^ | 28±5^*,#^ |
| **RMSSD (ms)** | 3.2±1 | 2.6±0.7 | 1.1±0.2^*^ | 1.4±0.3 | 16±3^*,#^ | 34±7^*,#^ |
| **CV (%)** | 2±0.5 | 1.3±0.3^ | 0.7±0.1^*^ | 1.3±0.3 | 1.3±0.3^*,#^ | 11±2*,#,^ |
| **pNN50 (%)** | 1±0.5 | 0±0^ | 0.1±0.1 | 0.1±0.1 | 2±1^*,#^ | 4 ±3^*,#^ |
| **ApEn** | 2±0.2 | 1.8±0.1 | 1.3±0.2^*^ | 1.5±0.2^#^ | 2.7±0.2^*,#^ | 2.8±0.3*,#,^ |
| **Frequency indices** | | | | | | |
| **VLF/Total (%)** | 21±3 | 15±3 | 13±2^*^ | 13±4 | 44±6^*,#^ | 34±6^*^,^#^ |
| **LF/Total (%)** | 12±3 | 36±8^ | 20±6 | 29±7 | 16±2 | 19±3^*,#^ |
| **HF/Total (%)** | 67±40 | 49±8^ | 67±9 | 58±6 | 40±5^*,#^ | 47±5 |
| **LF/HF** | 69±4 | 49±8^ | 67±9 | 50±8 | 61±10 | 62±13 |
| **Non-linear indices** | | | | | | |
| **β** | -1.3±0.2 | -1.7±0.1 | -1.8±0.1^*^ | -1.2±0.3#,^ | -1.9±0.3^*^ | -1.3±0.2*,^ |
| **α_1_** | 0.4±0.1 | 0.8±0.1^ | 0.3±0.1 | 0.8±0.2^ | 0.6±0.2 | 0.5±0.2^ |
| **α_2_** | 0.8±0.1 | 0.8±0.1 | 0.5±0.1^*^ | 0.7±0.1 | 0.8±0.1 | 0.7±0.1 |

**Table S3:** Measures of beating interval dynamics in vivo and in isolated tissue. SDNN, standard deviation of the inter beat interval; RMSSD, root mean square of the successive beat interval differences; CV, coefficient of variation of beat interval; pNN50, percentage of adjoining beating intervals differing by more than 50 ms; VLF, very-low frequency power; LF, low frequency power; HF, high frequency power; Total, total spectrum power; ApEn, approximate entropy; β, slope of the power-law relationship; α_1_ short-term scaling exponent; and α_2_ long-term scaling exponent. Beating intervals were recorded (i) in vivo in adult (3-4 months, n=17) and in aged (23-25 months, n=21) C57/BL6 mice and (ii) in isolated tissue from adult (3-4 months, n=21) and aged (23-25 months, n=21) C57/BL6 mice. *p<0.05 vs. basal in vivo, ^#^p<0.05 vs. intrinsic in vivo,^ p<0.06 vs. adult.

| **Parameter** | **Adult**  **Basal**  **In vivo** | **Aged**  **Basal**  **In vivo** | **Adult**  **Intrinsic**  **In vivo** | **Aged**  **Intrinsic**  **In vivo** |
| --- | --- | --- | --- | --- |
| **Time-domain indices** | | | | |
| **Beating rate (ms)** | 111±5 | 110±19 | 117±1 | 127±11 |
| **SDNN (ms)** | 16±2 | 7±4 | 4±2^*^ | 3±2 |
| **RMSSD (ms)** | 9±2 | 3±1^ | 2±0.3^*^ | 2±0.6 |
| **CV (%)** | 12±2 | 6±3^ | 3±1.7^*^ | 2±1 |
| **pNN50 (%)** | 0.2±0.2 | 0.1±0.1 | 0.1±0.1 | 0.1±0.1 |
| **ApEn** | 1.5±0.3 | 0.9±0.4 | 0.4±0.1^*^ | 0.4±0.3 |
| **Frequency indices** | | | | |
| **VLF/Total (%)** | 24±12 | 17±3 | 15±4 | 7±5 |
| **LF/Total (%)** | 19±3 | 17±4 | 11±9 | 20±5 |
| **HF/Total (%)** | 57±11 | 66±4 | 74±15 | 73±10 |
| **LF/HF** | 38±7 | 25±4 | 50±22 | 58±20 |
| **Non-linear indices** | | | | |
| **α_1_** | 1.1±0.1 | 1.2±0.1 | 0.9±0.1 | 0.8±0.2 |
| **α_2_** | 1±0.1 | 1.1±0.1 | 1.6±0.1^*^ | 1.2±0.1 |

**Table S4:** Measures of beating interval dynamics in vivo in awake mice. SDNN, standard deviation of the inter beat interval; RMSSD, root mean square of the successive beat interval differences; CV, coefficient of variation of beat interval; pNN50, percentage of adjoining beating intervals differing by more than 50 ms; VLF, very-low frequency power; LF, low frequency power; HF, high frequency power; Total, total spectrum power; ApEn, approximate entropy; α_1_ short-term scaling exponent; and α_2_ long-term scaling exponent. Beating intervals were recorded (i) in vivo in adult (3-4 months, n=3) and in aged (23-25 months, n=3) C57/BL6 mice. *p<0.05 vs. basal in vivo, ^#^p<0.05 vs. intrinsic in vivo,^ p<0.06 vs. adult.

| **(% change from control)** | **Adult** | | | | **Aged** | | | |
| --- | --- | --- | --- | --- | --- | --- | --- | --- |
|  | **ISO**  **1nM** | **ISO**  **10 nM** | **ISO**  **100 nM** | **ISO**  **1000 nM** | **ISO**  **1nM** | **ISO**  **10 nM** | **ISO**  **100 nM** | **ISO**  **1000 nM** |
| **Time-domain indices** | | | | | | | | |
| **Beating interval** | -15±10 | -32±12 | -45±12 | -43±14 | -6±3^*^ | -11±5^*^ | -65±10^*^ | -80±13 |
| **SDNN** | -37±14 | -65±9 | -67±7 | -61±12 | -27±12^*^ | -42±10^*^ | -72±8 | -83±6 |
| **RMSSD** | -2±8 | -66±6 | -69±8 | -66±9 | -18±5^*^ | -26±16 | -64±10 | -83±8 |
| **CV** | -30±14 | -40±13 | -55±9 | -57±8 | -18±10^*^ | -35±10 | -54±11 | -71±10 |
| **pNN50** | -29±31 | -62±20 | -68±14 | -63±20 | -23±29 | -65±12 | -70±16 | -82±16 |
| **ApEn** | -18±9 | -28±8 | -30±9 | -33±10 | -11±4^*^ | -18±6^*^ | -35±5 | -39±5 |
| **Frequency indices** | | | | | | | | |
| **VLF/**  **Total** | 2±20 | 100±98 | 106±99 | 91±93 | 105±99 | 118±90 | 176±82 | 165±93 |
| **LF/**  **Total** | -48±54 | -23±19 | -57±40 | -30±59 | -1±19 | -22±8 | -39±16 | -55±20 |
| **HF/**  **Total** | 31±11 | -31±12 | -32±14 | -25±10 | -9±19 | -2±34 | -20±37 | -45±27 |
| **LF/HF** | -41±38 | 106±46 | -65±17 | 58±26 | -46±50 | -9±23 | -99±14 | 10±24 |
| **Non-linear indices** | | | | | | | | |
| **β** | 23±8 | 36±23 | 50±20 | 54±19 | 8±3^*^ | 47±35 | 79±33 | 93±91 |
| **α_1_** | 21±9 | 28±12 | 45±18 | 55±21 | 14±6^*^ | 34±7 | 40±9 | 61±7 |
| **α_2_** | 44±17 | 60±20 | 76±18 | 95±39 | 13±7^*^ | 37±7 | 55±11 | 97±33 |

**Table S5:** Measures of beating interval dynamics in isolated SAN tissue in response to β-adrenergic receptor stimulation. SDNN, standard deviation of the inter beat interval; RMSSD, root mean square of the successive beat interval differences; CV, coefficient of variation of beat interval; pNN50, percentage of adjoining beating intervals differing by more than 50 ms; VLF, very-low frequency power; LF, low frequency power; HF, high frequency power; Total, total spectrum power; ApEn, approximate entropy; β, slope of the power-law relationship; α_1_ short-term scaling exponent; and α_2_ long-term scaling Beating intervals were recorded in adult (3-4 months, n=6) and aged (23-25 months, n=6) C57/BL6 mice. *p<0.05 vs. adult.

| **(% change from control)** | **Adult**  **time-control** | **Aged**  **time-control** |
| --- | --- | --- |
| **Time-domain indices** | | |
| **Beating interval** | -3±5 | 1±5 |
| **SDNN** | 16±17 | -16±12 |
| **RMSSD** | 16±15 | -9±11 |
| **CV** | 19±18 | -10±4 |
| **pNN50** | 3±11 | 4±11 |
| **ApEn** | 10±12 | -7±7 |
| **Frequency indices** | | |
| **VLF/**  **Total** | -13±12 | -8±14 |
| **LF/**  **Total** | -15±16 | -17±12 |
| **HF/**  **Total** | 29±18 | 18±10 |
| **LF/HF** | -10±6 | -8±10 |
| **Non-linear indices** | | |
| **β** | -4±3 | -5±7 |
| **α_1_** | -6±10 | -9±5 |
| **α_2_** | -8±9 | -1±3 |

**Table S6:** Time controls for measures of beating interval dynamics in isolated SAN tissue. SDNN, standard deviation of the inter beat interval; RMSSD, root mean square of the successive beat interval differences; CV, coefficient of variation of beat interval; pNN50, percentage of adjoining beating intervals differing by more than 50 ms; VLF, very-low frequency power; LF, low frequency power; HF, high frequency power; Total, total spectrum power; ApEn, approximate entropy; β, slope of the power-law relationship; α_1_ short-term scaling exponent; and α_2_ long-term scaling Beating intervals were recorded in adult (3-4 months, n=6) and aged (23-25 months, n=6) C57/BL6 mice. *p<0.05 vs. control.

| **(% change from control)** | **Adult** | | | | **Aged** | | | |  | |
| --- | --- | --- | --- | --- | --- | --- | --- | --- | --- | --- |
|  | **CCh**  **1nM** | **CCh**  **10 nM** | **CCh**  **100 nM** | **CCh**  **1000 nM** | **CCh**  **1nM** | **CCh**  **10 nM** | **CCh**  **100 nM** | **CCh**  **1000 nM** | | |
| **Time-domain indices** | | | | | | | | | |  |
| **Beating interval** | 11±3 | 29±15 | 46±7 | 71±4 | 7±2 | 15±6^*^ | 21±7 | 47±8^*^ |  | |
| **SDNN** | 71±29 | 53±22 | 57±28 | 165±27 | 24±20 | 35±16 | 95±17 | 154±77 |  | |
| **RMSSD** | 70±56 | 52±53 | 63±23 | 185±24 | 25±28 | 48±15 | 100±93 | 124±48 |  | |
| **CV** | 45±25 | 54±28 | 55±14 | 189±27 | 16±13 | 25±15 | 92±26^*^ | 245±90^*^ |  | |
| **pNN50** | 21±18 | 44±21 | 97±39 | 198±31 | 55±42 | 128±37 | 215±49 | 220±99^*^ |  | |
| **ApEn** | 22±8 | 23±9 | 29±10 | 34±10 | 5±17^*^ | 79±97 | 207±84^*^ | 243±84^*^ |  | |
| **Frequency indices** | | | | | | | | | |  |
| **VLF/**  **Total** | 31±23 | 39±47 | 61±47 | 78±33 | 47±92 | 39±28 | 32±21 | 34±23 |  | |
| **LF/**  **Total** | 55±18 | 57±39 | 63±38 | 91±38 | 91±73 | 98±43 | 23±13 | 65±43 |  | |
| **HF/**  **Total** | -72±16 | -88±16 | -42±40 | -80±17 | -65±46 | -86±87 | -54±34 | -130±39^*^ |  | |
| **LF/HF** | -21±11 | -12±13 | 15±3 | 22±15 | 60±21 | 101±87 | 131±15 | 272±21 |  | |
| **Non-linear indices** | | | | | | | | | |  |
| **β** | -12±4 | -32±11 | -43±8 | -47±7 | -22±6^*^ | -33±9 | -49±9^*^ | -54±9^*^ |  | |
| **α_1_** | -27±9 | -33±7 | -38±6 | -39±16 | -11±6 | -13±5 | -26±8 | -30±9 |  | |
| **α_2_** | -23±7 | -32±4 | -35±3 | -36±4 | -29±10 | -17±8 | -41±6 | -47±7 |  | |

**Table S7:** Measures of beating interval dynamics in isolated SAN tissue in response to muscarinic receptor stimulation. SDNN, standard deviation of the inter beat interval; RMSSD, root mean square of the successive beat interval differences; CV, coefficient of variation of beat interval; pNN50, percentage of adjoining beating intervals differing by more than 50 ms; VLF, very-low frequency power; LF, low frequency power; HF, high frequency power; Total, total spectrum power; ApEn, approximate entropy; β, slope of the power-law relationship; α_1_ short-term scaling exponent; and α_2_ long-term scaling Beating intervals were recorded in adult (3-4 months, n=6) and aged (23-25 months, n=6) C57/BL6 mice. *p<0.05 vs. adult.

| **(% change from control)** | **Adult** | | | | **Aged** | | | |
| --- | --- | --- | --- | --- | --- | --- | --- | --- |
|  | **IBMX**  **1μM** | **IBMX**  **10μM** | **IBMX**  **20μM** | **IBMX**  **100μM** | **IBMX**  **1μM** | **IBMX**  **10μM** | **IBMX**  **20μM** | **IBMX**  **100μM** |
| **Time-domain indices** | | | | | | | | |
| **Beating interval** | -18±9 | -31±8 | -46±11 | -54±10 | -13±10^*^ | -23±15^*^ | -30±17 | -65±10 |
| **SDNN** | -28±5 | -41±4 | -55±9 | -57±11 | -30±9^*^ | -30±8 | -60±11 | -65±8 |
| **RMSSD** | -26±9 | -36±8 | -54±10 | -55±10 | -17±11^*^ | -20±10 | -52±11 | -59±11 |
| **CV** | -19±6 | -33±6 | -46±9 | -51±8 | -26±7^*^ | -32±6 | -49±12 | -49±17 |
| **pNN50** | -57±11 | -84±8 | -79±11 | -83±9 | -27±13^*^ | -30±21^*^ | -65±14 | -83±11 |
| **ApEn** | -10±5 | -29±9 | -36±6 | -39±7 | -13±9^*^ | -25±10 | -28±10 | -32±9 |
| **Frequency indices** | | | | | | | | |
| **VLF/**  **Total** | -40±57 | -43±48 | -41±20 | -41±84 | -25±20 | 9±12 | -56±19 | -61±9 |
| **LF/**  **Total** | 12±23 | 22±15 | 3±11 | 14±26 | -57±36 | -43±23 | 48±29 | 101±60 |
| **HF/**  **Total** | 22±29 | 5±10 | 21±20 | 23±27 | 89±65 | 79±40 | 15±16 | -26±19 |
| **LF/HF** | -8±15 | 15±13 | -15±9 | -19±14 | -20±17 | 22±13 | 19±15 | 89±44 |
| **Non-linear indices** | | | | | | | | |
| **β** | 17±6 | 38±1 | 48±8 | 56±13 | 34±17^*^ | 46±25 | 64±5 | 63±15 |
| **α_1_** | 4±4 | 34±24 | 34±24 | 59±37 | 3±1^*^ | 17±8 | 47±12 | 69±23 |
| **α_2_** | 1±5 | 16±26 | 18±26 | 53±21 | 45±30^*^ | 68±23 | 73±26 | 78±24 |

**Table S8:** Measures of beating interval dynamics in isolated SAN tissue in response to phosphodiesterase inhibition. SDNN, standard deviation of the inter beat interval; RMSSD, root mean square of the successive beat interval differences; CV, coefficient of variation of beat interval; pNN50, percentage of adjoining beating intervals differing by more than 50 ms; VLF, very-low frequency power; LF, low frequency power; HF, high frequency power; Total, total spectrum power; ApEn, approximate entropy; β, slope of the power-law relationship; α_1_ short-term scaling exponent; and α_2_ long-term scaling Beating intervals were recorded in adult (3-4 months, n=6) and aged (23-25 months, n=6) C57/BL6 mice. *p<0.05 vs. adult.

**Fig. S1:** Age-associated changes in beating rate in both in vivo and in denervated SAN. *p<0.05 vs. 3 months, #p<0.05 vs. basal in vivo at the same age, ^ p<0.06 vs. intrinsic in vivo at the same age.

**Figure S2:**  Age-associated changes in (A) beating interval, (B) coefficient of variance (CV) and (C) low frequency (LF) to high frequency (HF) ratio in awake mice in vivo (n=3) and during autonomic blockade (DB).

**Fig S3:** Dose response of beating rate, coefficient of variance (CV) and approximate entropy (ApEn) in response to different degrees of (A) β-AR stimulation by isoproterenol (ISO), (B) CR stimulation by carbachol (CCh) or (C) PDE inhibition by 3′-isobutylmethylxanthine (IBMX) in isolated SAN tissue from adult (n=6) and aged (n=6) mice for each drug intervention.


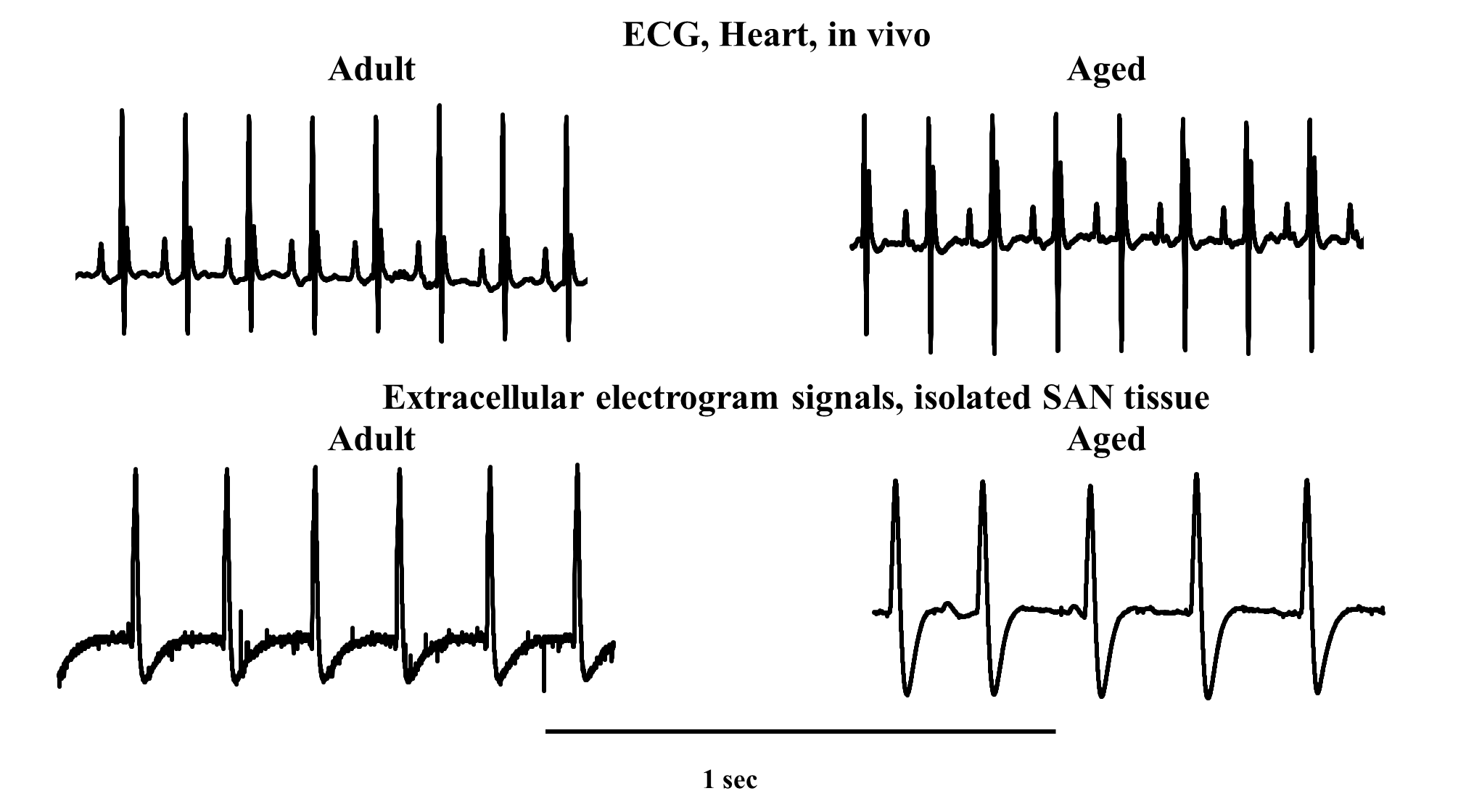


**Figure S1.**

**
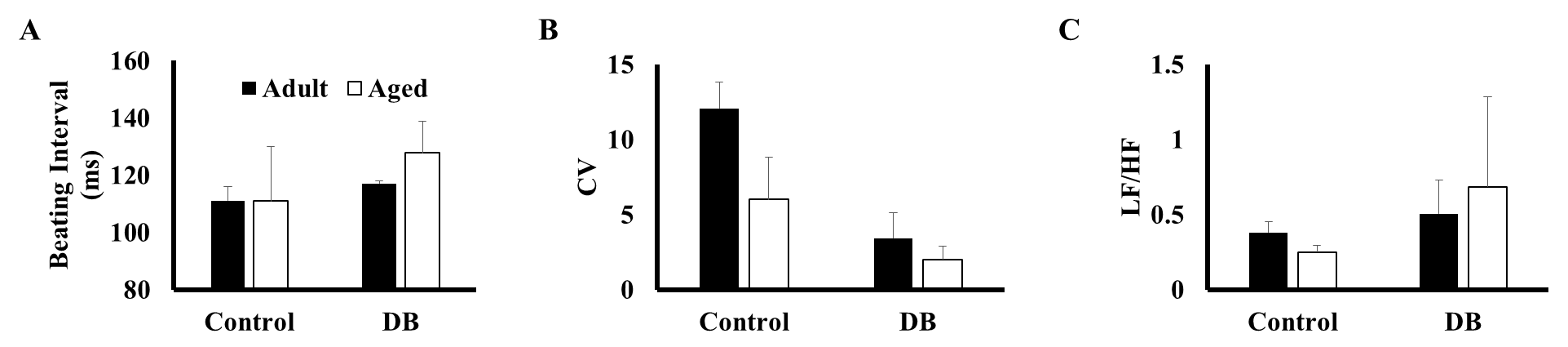
**

**Figure S2.**

**
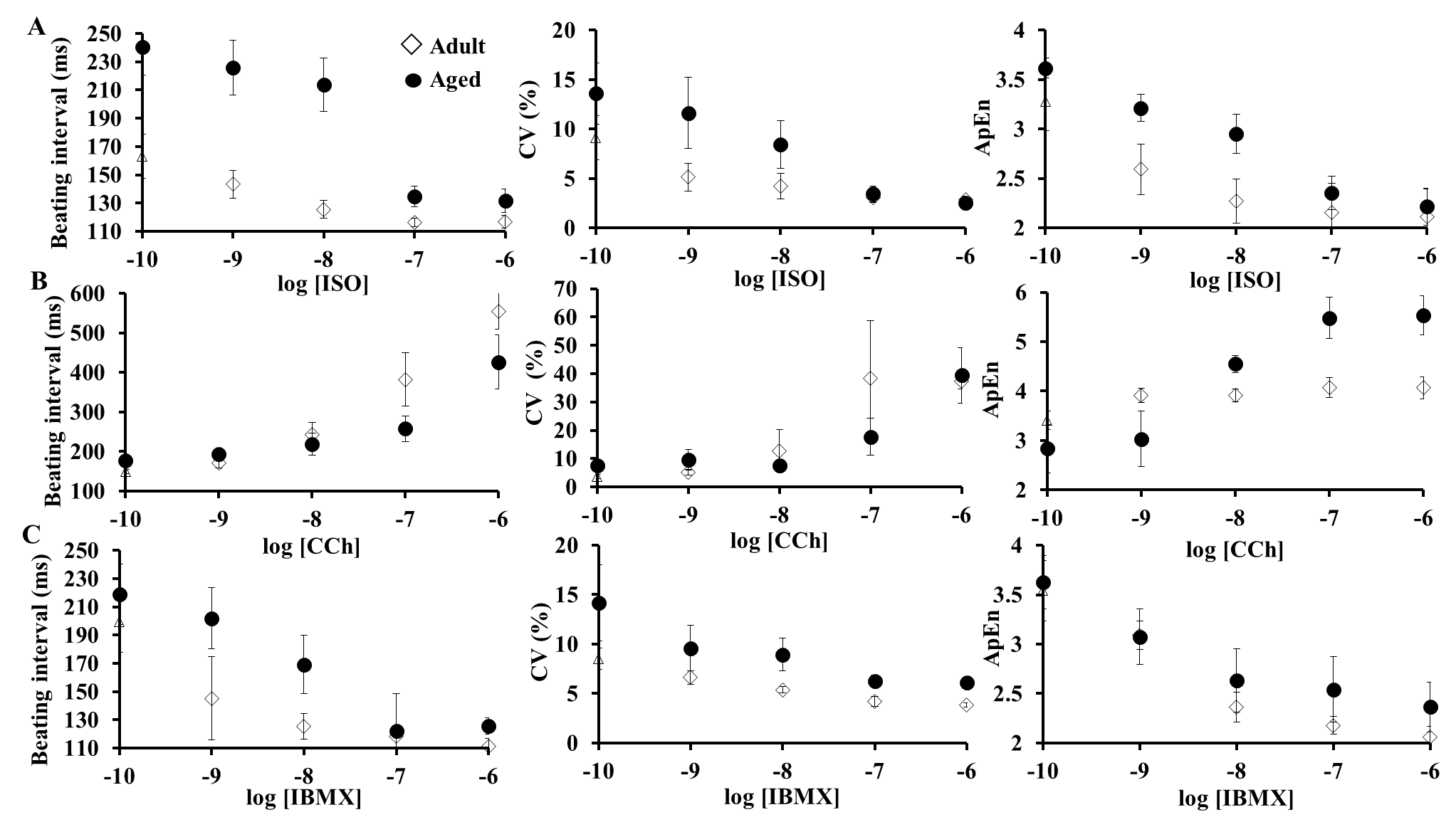
**

**Figure S3.**
